# Supplementary material for: Resolving the full spectrum of human genome variation using Linked-Reads
Source: Genome Res. 2019 Apr;29(4):635–45. doi: 10.1101/gr.234443.118 (PMC6442396; doi:10.1101/gr.234443.118)
Supplement: Supplemental Material [file supp_gr.234443.118_Supplemental_longranger-2.2.zip › longranger-2.2.tar.bz2!/lr2.2-public/lib/stan/cmdstan/stan/doc/api/html/math_2matrix_2dot__self_8hpp_source.html]

Stan: src/stan/math/matrix/dot\_self.hpp Source File


|  |  |
| --- | --- |
|  | Stan  2.5.0  probability, sampling & optimization |


- Main Page
- Related Pages
- Namespaces
- Classes
- Files

- File List
- File Members

All Classes Namespaces Files Functions Variables Typedefs Enumerations Enumerator Friends Macros Pages

- src
- stan
- math
- matrix

dot\_self.hpp

Go to the documentation of this file.

1 #ifndef STAN\_\_MATH\_\_MATRIX\_\_DOT\_SELF\_HPP

2 #define STAN\_\_MATH\_\_MATRIX\_\_DOT\_SELF\_HPP

3

4 #include <stan/math/matrix/Eigen.hpp>

5 #include <stan/math/error\_handling/matrix/check\_vector.hpp>

6

7 namespace stan {

8  namespace math {

9

17  template <int R, int C>

18  inline double dot\_self(const Eigen::Matrix<double, R, C>& v) {

19  stan::math::check\_vector("dot\_self(%1%)",v,"v",(double\*)0);

20  return v.squaredNorm();

21  }

22

23  }

24 }

25 #endif

stan::math::dot\_self

double dot\_self(const std::vector< double > &x)

**Definition:** dot\_self.hpp:11

stan::math::check\_vector

bool check\_vector(const char \*function, const Eigen::Matrix< T, R, C > &x, const char \*name, T\_result \*result)

**Definition:** check\_vector.hpp:14

Eigen.hpp

check\_vector.hpp


---

[ Stan Home Page ]

*© 2011–2014,
Stan Development Team.*
